# Cystic echinococcosis in South America: a call for action

**DOI:** 10.26633/RPSP.2017.42

**Published:** 2017-04-15

**Authors:** Carlos F Pavletic, Edmundo Larrieu, Eduardo A Guarnera, Natalia Casas, Pilar Irabedra, Ciro Ferreira, Julio Sayes, Cesar M Gavidia, Eduardo Caldas, Michael Laurence Zini Lise, Melody Maxwell, Marcos Arezo, Ana Maria Navarro, Marco A. N Vigilato, Ottorino Cosivi, Marcos Espinal, Victor J Del Rio Vilas

**Affiliations:** 1 Ministry of Health Ministry of Health Santiago Chile Ministry of Health, Santiago, Chile.; 2 Universidad Nacional de Rio Negro Universidad Nacional de Rio Negro Choele ChoelRio Negro Argentina Universidad Nacional de Rio Negro, Choele Choel, Rio Negro, Argentina.; 3 Instituto ANLIS-MALBRAN Instituto ANLIS-MALBRAN Buenos Aires Argentina Instituto ANLIS-MALBRAN, Buenos Aires, Argentina.; 4 Ministry of Health Ministry of Health Buenos Aires Argentina Ministry of Health, Buenos Aires, Argentina.; 5 Comisión Nacional Honoraria de Zoonosis Comisión Nacional Honoraria de Zoonosis Montevideo Uruguay Comisión Nacional Honoraria de Zoonosis, Montevideo, Uruguay.; 6 Universidad Nacional Mayor de San Marcos Facultad de Medicina Veterinaria Lima Peru Universidad Nacional Mayor de San Marcos, Facultad de Medicina Veterinaria, Lima, Peru.; 7 Secretary of Health Surveillance Ministry of Health Brasilia Brazil Secretary of Health Surveillance, Ministry of Health, Brasilia, Brazil.; 8 Ohio State University Ohio State University ColumbusOhio United States of America Ohio State University, Columbus, Ohio, United States of America.; 9 Ministry of Health Viedma ViedmaRio Negro Argentina Ministry of Health, Viedma, Rio Negro, Argentina.; 10 Ministry of Health Ministry of Health Lima Peru Ministry of Health, Lima, Peru.; 11 Department of Communicable Diseases and Health Analysis Pan American Health Organization, Regional Office of the World Health Organization Washington, DC United States Department of Communicable Diseases and Health Analysis, Pan American Health Organization, Regional Office of the World Health Organization, Washington, DC, United States.

**Keywords:** *Echinococcosis granulosus*, zoonoses, prevention & control, Argentina, Brazil, Chile, Peru, Uruguay, South America, *Echinococcus granulosus*, zoonosis, prevención & control, Argentina, Brasil, Chile, Perú, Uruguay, América del Sur, *Echinococcus granulosus*, zoonoses, prevenção & controle, Argentina, Brasil, Chile, Peru, Uruguay, América do Sul

## Abstract

Cystic echinococcosis (CE) or hydatidosis, a parasitic zoonosis caused by a cestode of the family Taeniidae, species Echinococcus granulosus, is endemic in Argentina, Chile, Peru, Uruguay, and southern Brazil. This report presents CE figures for these five countries in 2009 – 2014 and proposes indicators to measure national control programs.

Nearly 5 000 new CE cases were diagnosed annually in the five countries during the study period. The average case fatality rate was 2.9%, which suggests that CE led to approximately 880 deaths in these countries during the 6-year period. CE cases that required secondary or tertiary health care had average hospital stays of 10.6 days, causing a significant burden to health systems. The proportion of new cases (15%) in children less than 15 years of age suggests ongoing transmission.

Despite figures showing that CE is not under control in South America, the long-standing implementation of national and local control programs in three of the five countries has achieved reductions in some of the indicators. The Regional Initiative for the Control of CE, which includes the five countries and provides a framework for networking and collaboration, must intensify its efforts.

## RESUMEN

La equinococosis quística o hidatidosis, zoonosis parasitaria causada por un cestodo de la especie *Echinococcus granulosus*, familia Taeniidae, es endémica en Argentina, Chile, Perú, Uruguay y el sur del Brasil. En este informe se presentan datos sobre la equinococosis quística registrados en estos cinco países entre los años 2009 y 2014, y se proponen indicadores para medir el desempeño de los programas nacionales de control.

Durante el período de estudio, en los cinco países se diagnosticaron casi 5 000 casos nuevos por año. La tasa de letalidad promedio fue del 2,9 %, lo que indica que la equinococosis quística causó aproximadamente 880 defunciones en estos países en el período de 6 años estudiado. Los casos que debieron recibir atención de salud secundaria o terciaria tuvieron una estadía hospitalaria promedio de 10,6 días, lo que ha generado una carga significativa para los sistemas de salud. La proporción de casos nuevos de niños menores de 15 años (15 %) indica que la transmisión no se ha interrumpido. A pesar de que los datos muestran que la enfermedad no está controlada en América del Sur, tres de los cinco países disponen de programas nacionales y locales de control desde hace muchos años, los que han logrado reducir algunos de los indicadores. Es preciso que la Iniciativa Sudamericana para el Control de la Equinococosis Quística, que incluye a los cinco países y contribuye con un marco para la formación de redes y la colaboración, redoble sus esfuerzos para lograr el control de la enfermedad.

## RESUMO

A equinococose cística ou hidatidose, zoonose parasitária causada por um céstodo da família Taeniidae e espécie *Echinococcus granulosus*, é endêmica na Argentina, Chile, Peru, Uruguai e no sul do Brasil. Neste artigo são apresentadas as estimativas da hidatidose nos cinco países de 2009 a 2014 e propostos indicadores para avaliar os programas nacionais de controle.

Cerca de 5 mil casos novos de hidatidose foram diagnosticados por ano nos cinco países no período de estudo. A taxa de letalidade média foi de 2,9%, o que indica que esta doença causou aproximadamente 880 mortes nestes países no período de 6 anos. Nos casos de hidatidose que precisaram de atenção de saúde secundária ou terciária, a duração média da internação hospitalar foi de 10,6 dias, representando uma sobrecarga considerável aos sistemas de saúde. O percentual de novos casos (15%) em indivíduos menores de 15 anos de idade indica transmissão contínua. Apesar de as estimativas mostrarem que a hidatidose não está controlada na América do Sul, a execução permanente de programas municipais e nacionais de controle em três dos cinco países tem redundado na redução de alguns dos indicadores. A Iniciativa Regional para Controle da Hidatidose, que integra os cinco países em uma estrutura para trabalho em conjunto e colaboração, deve redobrar os seus esforços.

Cystic echinococcosis (CE) is a zoonosis of global distribution that results in important disability and mortality if not properly treated in a timely manner. CE is caused by *Echinococcus granulosus*, a cestode of the family Taeniidae whose hosts are herbivore and carnivore animals (1). The lifecycle of *E. granulosus* comprises three stages. First, the adult form lives in the small intestine of canine hosts and does not lead to any evident pathology, but produces large quantities of eggs. Second, the eggs are released into the environment in the feces, contaminating the soil and water. Humans and livestock become infected after ingestion of the eggs, either from direct contact with infected dogs or indirectly via contaminated water and food. In the third stage, the hydatid cyst (larval stage) develops in the viscera, mainly lungs and liver, of humans and herbivore animals resulting, for the latter, in reduced productivity. The death of an infected herbivore or its slaughter for human consumption, and the release of infected viscera into the environment, completes the parasite’s lifecycle if dogs have access to and eat the infected organ. In this context, unsupervised slaughter of herbivore animals constitutes the main route for disease transmission to the canine host (1, 2).

CE is considered a neglected disease with readily-available and effective tools for its control. The World Health Organization (WHO) has recommended implementing pilot projects in selected countries to validate the effectiveness and efficiency of CE control tools by 2015; and implementation of specific CE interventions, specifically in Latin America, to control and eliminate this public health concern by 2020 (3). Accordingly, the Pan American Health Organization (PAHO) considers CE to be a neglected disease that requires specific attention among poor and rural populations (4). This recognition followed earlier reports on the importance CE in South America (5–11).

In 2004, in response to requests by Argentina, Brazil, Chile, and Uruguay, PAHO coordinated the development of the Sub-Regional Project for the Surveillance and Control of CE. In 2013, Peru joined the project to constitute what is now known as the CE Initiative. The CE Initiative is comprised of heads of zoonosis departments/units within the Ministries of Health of the five countries, senior staff of Uruguay's Zoonoses National Commission, a ministerial-level department responsible for the implementation of CE control activities, senior officials of the Ministry of Agriculture of Peru, and a number of scientists and researchers from the five countries. The CE Initiative is currently coordinated by the zoonosis unit at the Pan American Center for Foot-and-Mouth Disease (PANAFTOSA, Rio de Janeiro, Brazil), a specialized center of PAHO. The CE Initiative is comprised of working groups focused on education activities, surveillance, control, and diagnostics.

The objective of the CE Initiative is to formulate strategies and action plans for CE control in South America. As a groundwork effort, the CE Initiative recently led a data collection effort to inform its first baseline on CE occurrence and program-specific indicators for five countries in South America. The objective of this work is to present this baseline data, introduce the CE Initiative’s activities, and discuss future opportunities for CE control.

## MATERIALS AND METHODS

In early 2015, with agreement from all members of the CE Initiative, PANAF-TOSA coordinated the data collection on all human and animal CE cases and program indicators for the period from 2009–2014. For Argentina, Brazil, Chile, and Peru, the information came from the CE program manager of each country’s Ministry of Health; for Uruguay, it was provided by the Zoonoses National Commission.

Each participating country was required to provide the following: year of CE control program initiation, annual budget, CE human case definition, number of CE human cases, average number of days in hospital, CE annual case fatality rate, and infection rates among those less than 15 years of age, as well as data on CE occurrence in livestock and canine echinococcosis. All of the information collected from these countries was found in local reports, if available, or in hospital registries. Data on livestock infection was complemented with information from the World Organization for Animal Health (Paris, France; OIE) for 2005– 2014. This report presents estimates of CE disease frequency and summary statistics of these program indicators, by country and aggregated at the sub regional level.

## RESULTS

### Program indicators

Human CE is a notifiable disease in Argentina, Chile, Uruguay, and in only one state of Brazil (Rio Grande do Sul). It is not notifiable in Peru.

In Argentina, CE has been notifiable since 1960 (National Law No. 15465); in Chile, since 1985 (Decree No. 11/85); and in Uruguay, since 1939 (Law No. 9852). Both Argentina and Uruguay have national surveillance and control programs. For Argentina, the 2014 budget was US$ 365 000, which covered procurement of dog and human anti-parasitic drugs, canine surveillance, ultrasound screening of school-children, and training of CE national field staff. In Chile, there is no national program, and therefore, no national budget for CE control, but such programs do exist in areas where the disease is endemic. For Uruguay, the 2014 budget of US$ 856 000 is coordinated nationwide by the Zoonoses National Commission and includes control activities for other zoonoses.

The countries have different case definitions (Table 1). Chile is the only one that has incorporated serology into the definition of a suspected case; the most complete confirmed case definition is that of Argentina, as it includes serology and imaging diagnostics. Uruguay is the only country that considers probable cases. Neither Brazil nor Peru have human CE case definitions.

### CE in humans

Data from two sources were used to inform the epidemiological indicators shown in this section: first, the network of outpatient centers reporting to a national surveillance system in Argentina, Brazil, Peru, and Uruguay; and second, patients discharged from the national hospital network in Chile.

**TABLE 1.** Case definitions for human Cystic Echinococcosis (CE) in three countries in South America where it is a notifiable disease, 2016

Country	Suspected case	Probable case	Confirmed case
Argentina	Two definitions: (i) A symptomatic or asymptomatic individual with a cystic mass (unique or multiple) localized in abdomen, trunk or elsewhere, and with epidemiology consistent with exposure to CE (e.g., place of origin, contact with dogs, relatives with history of CE), or (ii) An individual with a positive ultrasound.	Not defined	Any suspect case with positive imaging diagnostic tests (ultrasound, radiology, and/or computed tomography), and/or positive serology (ELISA, Western Blot, or HAI), or direct microscopic identification of the cestode parts (membranes, hooks, and protoscolex)
Chile	An individual with compatible clinical epidemiology consistent with exposure to CE, positive ultrasound, and/or serology	Not defined	Any suspected case with positive result to histopathology study or direct microscopic identification of the cestode (protoscolex)
Uruguay	An individual with one or more cystic masses in internal organs, mainly liver and lung, either asymptomatic or with manifestation via expansion, compression, occlusion, allergic reaction and/or infection, and with compatible epidemiology	Suspected case plus positive ultrasound and/or serology.	Any suspected case with positive histopathology, or direct microscopic identification of the cestode (protoscolex, hooks and/or membranes)

***Source:*** Prepared by the authors from the published data referenced by this study.

**FIGURE 1.**
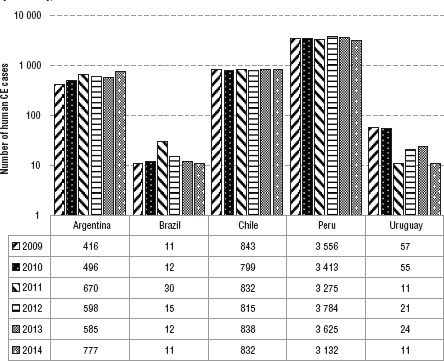
Number of cases of cystic echinococcosis (CE) in humans in South America, by country, 2009 – 2014

***Source***: Prepared by the authors from the published data referenced by this study.

**FIGURE 2.**
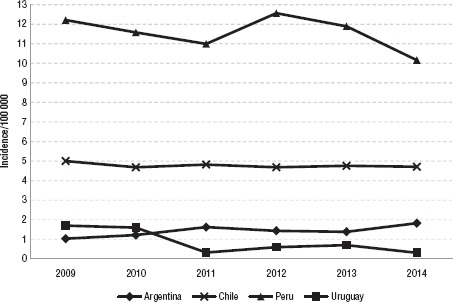
Annual incidence of cystic echinococcosis in humans (per 100 000) in four countries in South America, 2009 – 2014

***Source***: Prepared by the authors from the published data referenced by this study.

From January 2009–December 2014, a total of 29 559 new human cases of CE were registered by the five countries. Peru reported 20 785 cases, followed by Chile with 4 959, Argentina with 3 542 cases, Uruguay with 179, and Brazil with 94. Details by year and country are shown in Figure 1. Annual incidences per 100 000 population for Argentina, Chile, Peru, and Uruguay are shown in Figure 2.

**FIGURE 3.**
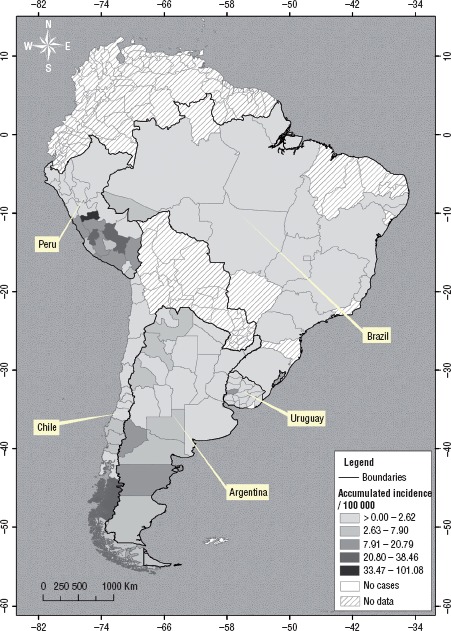
Map of cumulative human cystic echinococcosis incidence by country in South America, 2009 – 2014

***Source***: Prepared by the authors from the published data referenced by this study.

To depict the heterogeneous distribution of the disease within the countries, Figure 3 shows the cumulative incidence per 100 000 population by first-order administrative unit (provinces in Argentina, >departments in Uruguay, regions in Chile and Peru) from 2010–2014 for all countries except Brazil, which could not provide this level of detail. The denominator was the mid-year population in 2012. Argentina shows three areas where the incidence is high: the Patagonia in the south (the provinces of Neuquén and Chubut with the highest rates), the northwest (including the provinces of Catamarca, Santiago del Estero, and Salta), and the eastern province of Entre Rios. In Southern Chile, Bio Bio, La Araucania, Los Rios, Los Lagos, and Aysen areas had the highest rates, while Magallanes showed increased incidence. In Peru, the highest incidence was in the Central and South Highlands, specifically, Arequipa, Cusco, Huancavelica, Junín, Pasco, and Puno. In Uruguay, CE incidence occurs in the northwest and central areas. In Brazil, although not shown on the map, CE is mostly detected in the states of Acre and Rio Grande do Sul.

The average case fatality rate (CFR) across the five countries was 2.9%. This suggests that CE led to approximately 880 deaths in the five countries during the 6-year period. Argentina reported an annual average CFR of 2.7% in 2009– 2013, and no evident trend was observed. Chile reported an average annual CFR of 0.72% for the 4 years from 2009–2012. Brazil reported just 3 years of data, from 2011–2013, and presented the highest average CFR at 7.2%. Both Peru and Uruguay provided only 1 year of data, with 1.9% and 2.0% CFR, respectively.

Two other indicators were collected: (i) the proportion of CE cases reported among children less than 15 years of age—indicative of a persistent environmental risk leading to new cases among children; and (ii) the average number of days spent by CE patients in hospital—a potential proxy for underlying indicators, such as the severity of cases presenting to the health system (related to the timeliness of the surveillance mechanisms) and the impact of new treatment approaches or health care policies on patient time in hospital. The average proportion of cases among children less than 15 years of age was 15.0%. Averaged across the study period, 15.8% of cases in Argentina were in children 15 years of age or younger, 18.8% in Brazil, 15.1% in Chile, 17.04% in Peru, and 6.45% in Uruguay.

All countries except Peru had data for the annual average number of days spent by CE patients in hospital, though not for all years. The average for the four countries was 10.6 hospital-days. Country- specific averages were 9.8 days for Argentina, 11.0 for Brazil, 12.2 for Chile, and 8.7 for Uruguay.

### CE in animals

CE in animals is a notifiable condition to the OIE. Data on annual CE cases in 2005–2014, as detected after post-mortem inspection at abattoirs and reported by the veterinary authorities of the five countries, was requested from the OIE.

More than 9.5 million CE cases in livestock were reported by the five countries. Cattle accounted for 80% of the total, ovine for 13%, and swine for 6%. Other animal species, such as equine, caprine, and South American camelids were also reported, although in much smaller numbers. The data shows a decline in the number of cases in all species from 2010–2014; however, this needs to be interpreted with caution due to a number of possible surveillance issues.

To complement the OIE figures, the countries were asked to provide CE prevalence estimates based on abattoir post-mortem inspections. These figures were provided directly by the countries to the authors, using the same spreadsheet format as used for human cases (Table 2). Not all the countries could provide data for all years, e.g., Uruguay provided prevalence estimates in cattle for all years except 2012, and Argentina did not provide estimates for sheep.

To complement the data on CE cases in humans and livestock, results of all studies of dogs from all countries were also requested. However, diagnosis of Canine

**TABLE 2.** Cystic echinococcosis occurrence in ovine and bovine in five countries in South America, by country and year, 2009–2014

Year	Argentina		Brazil		Chile		Peru		Uruguay	
	Ovine (%)	Bovine (%)	Ovine (%)	Bovine (%)	Ovine (%)	Bovine (%)	Ovine (%)	Bovine (%)	Ovine (%)	Bovine (%)
2014	– ^a^	–	4.64	0.27	–	–	–	–	2.2	3.9
2013	–	0.43	3.12	0.29	2.1	15.0	–	–	3.6	5.7
2012	–	0.44	8.65	0.35	3.0	17.8	6.55	6.12	–	–
2011	–	0.49	11.15	0.44	2.3	12.6	0.28	4.95	2.2	5.5
2010	–	0.55	12.04	0.45	1.9	14.5	10.44	3.61	5.8	6.9
2009	–	0.40	8.76	0.37	1.4	15.2	9.48	4.85	5.9	7.05

a No data available.

***Source:*** Prepared by the authors from the published data referenced by this study.

**TABLE 3.** Cystic echinococcosis prevalence in dogs, by country and year, with detail of the locations where surveys were conducted using in-house copro-ELISA techniques, Argentina, Uruguay, and Peru

Year	Argentina		Uruguay		Peru	
	Prevalence^a^ %	Study locations	Prevalence %	Study locations	Prevalence	Study locations
2014			1.8	Nationwide		
2013	13.4 [28/209]	La Rioja	1.6		42.1	Junin, Pasco, Puno
2012	19.3 [22/114]	Tucuman, Jujuy, La Pampa	5.2			
2011	11.7 [38/324]	Tucuman, Jujuy	3.8			
2010	5.7 [31/541]	San Luis, Jujuy	4.3			
2009	17.7 [20/113]	Cordoba, Entre Rios	3.2			

a In brackets, number of positive CE samples of total collected.

***Source:*** Prepared by the authors from the published data referenced by this study.

Echinococcosis is not systematically carried out by all the countries, and different studies targeted different at-risk areas throughout the years, making interpretation of results difficult. Neither Brazil nor Chile reported data on these indicators (Table 3). The highest prevalence, 42.1%, was observed in the Central Highlands of Peru.

## DISCUSSION

CE is an important public health concern in South America, as shown by these results and significant underestimations of the real burden of disease. Assuming that underestimation has been unchanged in recent years—since CE notification and registration have not improved—our figures show a significant increase over the estimated 2 000 new cases per year that were previously reported (12). Like other neglected conditions, CE surveillance suffers from severe under-reporting and some sources estimate that its true incidence could be 2–100 times greater than reported (13).

Based on the number of cases, CE is the most important zoonosis in Argentina (1). In Peru, with more than 3 000 human cases reported annually and areas presenting annual incidence estimates of more than 100 cases/100 000 (Figure 3), the burden of disease is unquestionable (9, 11). In Peru, Moro and colleagues (14) reported annual disability-adjusted life years (DALYs) for CE as high as those of malaria and amoebiasis, using for their analysis the 2 189 human CE cases recorded in 2007 by the Ministry of Health. This number implies more than 3 400 cases per year from 2009—2014 and would significantly increase the DALYs if a new study were conducted today.

In Argentina and Chile, CE remains a problem in rural and poor communities. In both cases, incidence estimates have remained stubbornly stable for the 2009– 2014 period (Figure 2). In Uruguay, most of the epidemiological indicators show a declining trend.

Previous reviews of the situation in South America (12) reported the need for a long-term, political and financial commitment to support a widespread CE control program. The same report also commented on the failure of reaching CE elimination in the sub region, despite the existence of readily available tools (4). Although some of the countries in South America boast longstanding CE control efforts (Uruguay since 1970; Argentina, 1980; and Chile, 1982), our results do not show considerable progress towards control, except for in Uruguay and localized successes elsewhere (13, 15). On the other hand, Peru has just started pilot control projects in five endemic parts of the country. And yet, Brazil remains without a national coordinated effort, though there are incipient initiatives towards enhanced data collection and coordination.

An established CE case definition exists in only three of the five study countries (Argentina, Chile, and Uruguay) and these differ from one another; and despite no official case definition, Brazil and Peru are registering CE cases (Table 1). All countries except Chile, report notified cases, usually from outpatient centers where parasitological confirmation is not possible. Chile, on the other hand, reports hospitalized cases, which include parasitological confirmation; this system is therefore more specific than others that rely on serological, ultrasound, and/or clinical diagnosis. Hospital-based surveillance might be less sensitive than serology, ultrasound, and/or clinical diagnoses, which can detect asymptomatic cases as well as symptomatic ones that do not present to the hospital. The need to develop a standard case definition for all countries is obvious; it is one of the CE Initiative goals.

Further work is also necessary to qualify the canine prevalence estimates reported by Argentina, Peru, and Uruguay so that fair comparisons may be drawn. The three countries provided estimates by year that aggregated different localities at greater risk of CE. The 42% prevalence estimate reported by Peru is comparable to early estimates from other settings in South America at the start of their control programs (13). However, there is a need for a better understanding of the definitions and protocols that underpin sample collection and aggregation.

In addition, different diagnostic techniques among the countries may further confound comparisons. This may be the case, for example, with dog prevalence estimates. For instance, Argentina and Uruguay reported using the ELISA copro-antigen technique, but it is unclear whether the same assay or protocols were used, and even if so, whether there were modifications that could hamper comparisons. For this reason, the CE Initiative, as part of the diagnostic working group’s activities, is collecting precise information on protocols from the national reference laboratories. This data will inform the forthcoming results of a laboratory proficiency exercise.

Regarding CE in animals, similar precautions should be taken when interpreting the data (16). The decrease observed in reported CE cases is likely to reflect surveillance artifacts rather than disease dynamics. Changes in the number of reporting abattoirs or modifications in post-mortem practices were reported by members of the CE Initiative as the most likely predictors of the observed trends. In order to support robust comparisons between countries, and even within countries over time, there is clearly a need to map out the processes related to post-mortem inspection at abattoirs.

### The value of a regional initiative

CE control is the responsibility of national and local government, but past success with other diseases, e.g., rabies, shows that a sufficiently resourced, longterm regional program can benefit national control efforts. A regional program increases awareness and promotes the exchange of best practices. The CE Initiative, core-funded by PAHO/WHO with ad-hoc funds from local organizing committees, supports members’ attendance at conferences and networking. With an annual core-budget of around US$ 18K and continuous commitment from its members, the outputs delivered by the CE Initiative show great efficiency and value.

Since 2004, the CE Initiative has delivered on a number of fronts (17). At the onset, it supported technical cooperation among countries via specific projects: (i) laboratory training for laboratory personnel in Bolivia and Peru by officials from Uruguay; (ii) coordinated control efforts between Argentina and Chile in Tierra del Fuego; (iii) and strengthened control activities, such as trainings and public awareness between Brazil and Uruguay at their shared border. Recent activities have included the development of an online course given by a large consortium of academics and officials (18); the first epidemiological report of CE in the Americas (19); the first proficiency exercise for national reference laboratories; in-situ trainings of laboratory personnel; periodic conference calls; and in-situ meetings, among others.

For the biennium from 2016–2017, the CE Initiative plans to focus on advocacy activities and on the evaluation of national CE control programs. This report supports the first goal. A second epidemiological report on CE program indicators and occurrence in the South America is planned for release in mid-2017. Future epidemiological reports and outputs like this will amend the lists of indicators to allow comparisons with earlier works. For example, here we report on the proportion of cases in children less than 15 years of age, whereas Larrieu and Zanini (12) reported on incidence among those less than 14 years of age. In addition, the new information template will include data fields to capture treatment protocols from across the countries.

As for the evaluation of control programs, the CE Initiative has developed an exhaustive evaluation tool comprising all the capacities that constitute a standard CE control program. The tool, that also provides a number of indicators to monitor progress towards control, is based on similar frameworks developed for other diseases (20) and will allow the strategic evaluation of capacities, identification of gaps, and guidance for prioritization. The tool was applied first in Uruguay in mid- 2016. Future application of the tool in the other countries will allow for a thorough assessment of the regional capacity, will support country-specific plans for CE control, and as a result, will inform the goals of the CE Initiative as it strives toward control.

### Opportunities for improved surveillance and control

This report presents CE occurrence figures for humans and animals separately. Efforts are required to formally integrate these figures so that CE risk to humans can be comprehensively and prospectively informed. Recent studies that show the formal integration of syndromic surveillance data to predict occurrence of cases among a population of interest may merit further study for CE (21). This concurs with the recommendations of the 2012 Inter-American Ministerial Meeting on Health and Agriculture (22) that advocated for approaches to integrating animal health with public health surveillance data.

Though the goal is for all countries to be represented at the CE Initiative by their official veterinary sector and/or Ministry of Agriculture, currently only Argentina, Peru, and Uruguay have such representation. Increased participation of national veterinary authorities would bring clear benefits, shedding light on the biases underlying CE reporting in livestock, promoting discussions to correct the sources of such, and ultimately, contributing solutions and resources to control CE at its origin.

In addition, cooperation and integration for CE control should not stop at the Ministry of Health and Ministry of Agriculture; rather a wider net should be cast to include other governmental departments and the private sector, including corporations. Public-private partnerships have been reported to be a critical element in the effective implementation of control efforts for other diseases (4) and should be further explored for CE.

### Conclusions

The CE Initiative has been successful in promoting and developing strategies and action plans for control of this public health threat in South America. In July 2016, the 17th Inter-American Ministerial Meeting on Health and Agriculture (23) focused on the theme “One Health and the Sustainable Development Goals.” Representatives drew up a 2016–2022 action plan that calls for the elimination of neglected, infectious diseases in South America. The plan was subsequently presented to the PAHO Governing Bodies. There are undoubtedly, clear opportunities for increasing the profile of CE and advancing efforts for its control within strategic regional programs.

#### Acknowledgements.

The members of the CE Initiative wish to thank the staff of the Ministry of Health and Ministry of Agriculture in each of the five participating countries who collaborated in obtaining the data for this report. We also thank PANAFTOSA (Rio de Janeiro, Brazil) for its support and coordination with both the meeting planning and manuscript production.

#### Disclaimer.

Authors hold sole responsibility for the views expressed in the manuscript, which may not necessarily reflect the opinion or policy of the *RPSP/PAJPH* and/or PAHO.
